# Development of Location-Data-Based Orchard Passage Map Generation Method

**DOI:** 10.3390/s24030795

**Published:** 2024-01-25

**Authors:** Joong-hee Han, Chi-ho Park, Young Yoon Jang

**Affiliations:** 1 Division of Electronics and Information System, Daegu Gyeongbuk Institute of Science and Technology (DGIST), Daegu 42988, Republic of Korea; jhhan@dgist.ac.kr; 2 Sungboo IND Ltd., Chilgok-gun 39909, Republic of Korea; chang1y@hanmail.net

**Keywords:** path generation, orchard passage map, waypoints, autonomous driving, speed sprayer

## Abstract

Currently, pest control work using speed sprayers results in increasing numbers of safety accidents such as worker pesticide poisoning and rollover of vehicles during work. To address this, there is growing interest in autonomous driving technology for speed sprayers. To commercialize and rapidly expand the use of self-driving speed sprayers, an economically efficient self-driving speed sprayer using a minimum number of sensors is essential. This study developed an orchard passage map using location data acquired from positioning sensors to generate autonomous driving paths, without installing additional sensors. The method for creating the orchard passage map presented in this study was to create paths using location data obtained by manually driving the speed sprayer and merging them. In addition, to apply the orchard passage map when operating autonomously, a method is introduced for generating an autonomous driving path for the work start point movement path, work path, and return point movement path.

## 1. Introduction

A speed sprayer is an agricultural machine used for pest control work. Existing speed sprayers are prone to spraying accidents, leading to pesticide poisoning of workers, and safety accidents while driving, when rollovers occur. In addition, pest control work is the most laborious among the various aspects of fruit farming, and it is difficult to find hired labor.

To address these problems, research has been conducted to develop a speed sprayer capable of autonomous driving. Cantelli et al. [1] proposed an autonomous vehicle and a spraying management system to allow safe and accurate autonomous spraying operations, using a stereo camera, a global navigation satellite systems (GNSS) receiver, an attitude and heading reference system (AHRS), a laser scanner rangefinder, ultrasonic sensors, and motor encoders. In Zhang et al. [2], to improve the accuracy and reliability of autonomous driving of orchard spraying robots, an integrated navigation system was developed, consisting of a real-time kinematic positioning–Beidou satellite navigation system (RTK-BDS) receiver, an inertial measurement unit (IMU), a navigation controller, and servo motors. In Yu and Song [3], a remote driving system for a robotic speed sprayer designed to operate autonomously was proposed to aid agricultural machines. This paper evaluated the performance of a remote driving system for a robotic speed sprayer based on a combination of three types of sensors. It employed a surround-view monitoring system, a line-tracking device and medium-angle camera, and a line-tracking device and a wide-angle camera. In Wang et al. [4], an autonomous orchard navigation spray system was developed using laser radar and millimeter-wave radar sensing technology. In Qin et al. [5], a map-based collaborative navigation system for autonomously driven spraying–dosing robot groups was developed using navigation sensors, including a GNSS receiver and light detection and ranging (LiDAR).

To perform successfully in an orchard, autonomous driving agricultural machinery needs to follow a desired trajectory, and an autonomous driving path needs to be defined for that purpose. Autonomous driving path definition methods for orchards can be classified into four types, based on previous studies. The first method is to define an autonomous driving path based on georeferencing aerial images [6,7]. This method uses aerial images with high resolution and location accuracy, making it possible to check the locations of all passages and trees in the orchard, making it easy to plan a path for the autonomous operation of multiple agricultural machines at the same time. However, there is a disadvantage, in that additional costs are required to construct the georeferencing aerial images. 

The second method is to detect the ends of rows of fruit trees and then generate an autonomous driving path [8,9]. Since this method only obtains information about the ends of rows of fruit trees, it has the advantage of being able to obtain information quickly and create a path even when the orchard is large. However, since the path definition of the turning section uses a vehicle model, there is a risk of collision in narrow turning sections. In addition, if the tree line is not straight, there is a risk of collision with trees in a supposedly straight section. Another problem is that an incorrect path may be created when an inaccurate location is obtained due to the performance of the LiDAR or the camera, the sensor that detects the ends of the rows of fruit trees, or environmental factors. 

The third method is to construct a map of a local orchard based on tree trunk detection using cameras and/or LiDAR [4,5,10]. This method is advantageous because it generates a map suitable for autonomous driving while driving through an orchard passage using a mapping vehicle or autonomous agricultural machinery. Additionally, when SLAM technology is applied, the map can be updated to reflect the latest situation, even if the orchard environment changes. However, the LiDAR or camera may obtain inaccurate data due to poor weather or illumination, so the locations of objects may be mapped incorrectly in the generated map. 

The fourth method is to obtain location data for the path on which autonomous driving will be conducted and define the path [11,12]. This method is cost-effective when creating a path because it can establish a path for autonomous agricultural machinery equipped with only low-cost positioning sensors such as GNSS. However, since only the location of the path is known, and not the trees, there is a risk of collision during autonomous driving operation.

To promote the commercialization and expansion of self-driving speed sprayers used in orchards, it will be necessary to develop a self-driving speed sprayer with minimal sensors and price competitiveness. In particular, since most of the autonomous driving speed sprayers currently being studied are based on positioning sensors such as GNSS, the autonomous driving path generation method using location data is expected to be highly useful as it can be applied without additional sensors. Based on the two perspectives mentioned above, this study applied the path generation method using location data, which has the following advantages among the route generation methods: First, since the location data obtained by manually driving the speed sprayer are used, there is no additional cost incurred when creating an orchard map. In addition, since the speed sprayer travels only through pre-determined passages, it does not require information on the entire passageway in the orchard and only needs to obtain location data by manually driving only some passages in the orchard. Compared with cameras or LiDAR, the data capacity is very small, so data processing is possible on a low-cost embedded computer that does not include a graphics processing unit (GPU).

Therefore, in this study, a method of generating an orchard passage map using location data and a method of generating an autonomous driving path using an orchard passage map are presented for an autonomously driven speed sprayer. Then the presented method was verified by conducting experiments in an orchard. The descriptions of the orchard passage map data generation method and the autonomous driving path generation method are presented in Section 2. Test results of the proposed methods are provided in Section 3. The conclusion and future work are given in Section 4.

## 2. Methods

### 2.1. Orchard Passage Map Data Generation

Orchard passage map data consisted of work passages, location points for all orchard passages, network data, and spraying working methods and were created by acquiring location data for individual paths, creating waypoints for the individual path, and then merging them. To obtain location data, a positioning sensor that can acquire the geodetic position of the speed sprayer to be driven manually must be installed. The positioning sensor must provide a position with centimeter-level accuracy, and positioning sensors that can be used to acquire location data include GNSS positioning sensors or GNSS and inertial measurement unit (IMU) fusion positioning sensors. Orchard passage map data were created through post-processing using location data acquired through manual driving.

#### 2.1.1. Individual Path Generation

**Figure 1:**
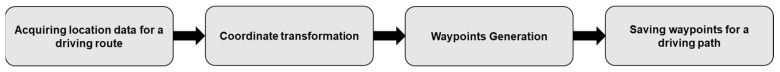
Process for the individual path generation.

Individual path generation was used to create waypoints by obtaining location data on a driving path in an orchard by manual driving. The types of individual paths defined in this study were divided into working and auxiliary paths. The working path was a driving path in which the speed sprayer performed spraying while sequentially driving through the orchard passages. The auxiliary path was a driving path that the speed sprayer could drive on among the orchard passages but which excluded the working path. The individual path generation process is shown in Figure 1. 

The first step in individual path generation was to acquire location data for a driving path, by manually driving a speed sprayer. The location data for generating a working path were obtained by manually driving the speed sprayer sequentially from the point where work began to the end point, along the orchard passages. The data to create an auxiliary path were obtained by driving along the orchard paths, excluding the working path. 

**Figure 2:**
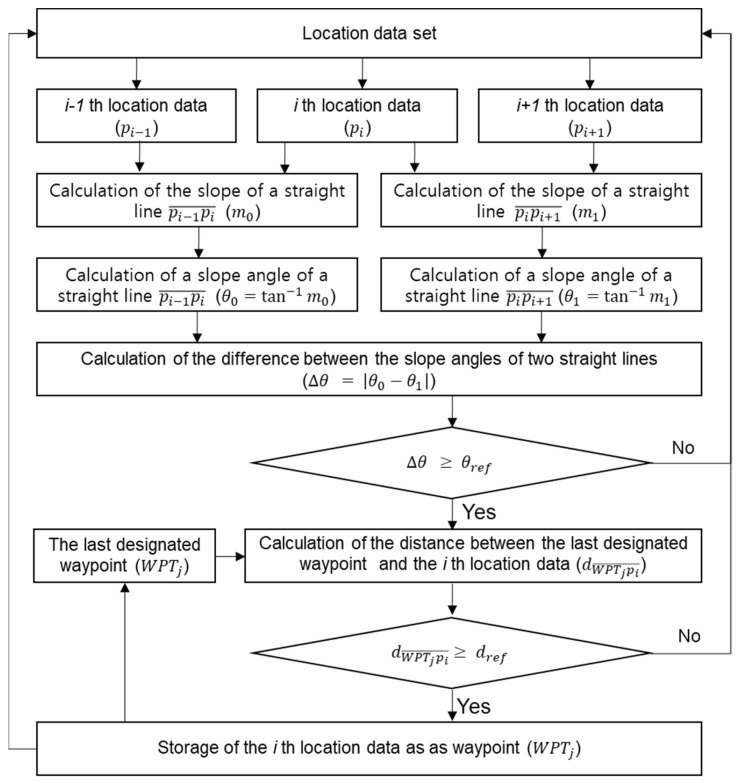
Waypoint generation process.

Next, since the acquired position data were geodetic coordinates on an ellipsoid, they were converted to north-east-down (NED) coordinates, which are positions in the local tangent plane coordinates. In the waypoint generation step, some of the location data acquired through manual driving were designated as waypoints along the autonomous driving paths. Waypoints were generated using the method shown in Figure 2, based on the minimum distance between waypoints ($d_{ref}$), which was predefined by the user as the standard value for generating waypoints, and the angle between straight lines ($\theta_{ref}$), which determined whether or not they were straight. Last, the geodetic coordinates of the location data designated as the waypoints were saved.

#### 2.1.2. Orchard Passage Map Data Generation

**Figure 3:**
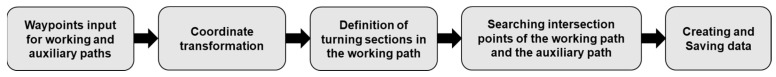
Orchard passage map data generation process.

The orchard passage map data generation process was used to create waypoints for work passages, location points for all orchard passages, network data, and spraying working methods in work passages, using waypoints for the working and auxiliary paths generated in the individual path generation step. Figure 3 shows the process for orchard passages map data generation.

In order to generate orchard map data, the first step was to input waypoints for both working and auxiliary paths, which were generated by the individual path generation. In this step, one entire working path waypoint and one or more auxiliary path waypoints were input. Next, since the locations of the input waypoints were expressed in geodetic coordinates, they were converted to NED coordinates for convenience of calculation. 

Since auxiliary paths included turning sections where the speed sprayer drove into an adjacent work passage in the orchard, in the third step of the orchard passage map data generation, turning sections were defined in the working path before merging the working path and the auxiliary paths. The process of defining turning sections was as follows: A rotation waypoint was designated when the angle between two straight lines created by adjacent front and rear waypoints was greater than a certain angle. Next, the distance between adjacent rotation waypoints was calculated, and rotation waypoints that were greater than a certain distance were extracted. Last, the start and end waypoints of each turning section were specified using the waypoints extracted in the previous step and the first and end waypoints of the working path.

**Figure 4:**
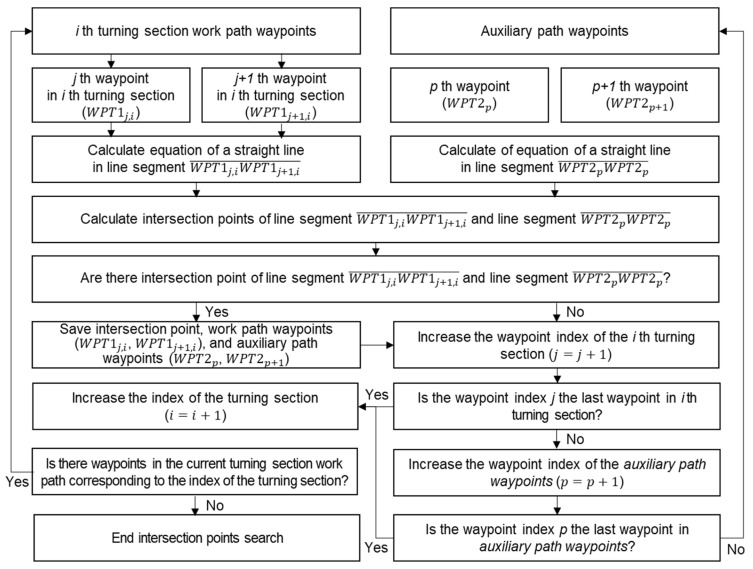
The line-segment-based intersection points search process.

The third step of the orchard passage map data generation was to search for intersection points of the working path and the auxiliary path. The first search method for intersection points was the line-segment-based intersection point search. It attempted to determine whether there was an intersection point between the line segment created as the work path’s waypoints for each rotation section and the line segment created as the auxiliary path’s waypoints. The line-segment-based intersection point search process is shown in Figure 4.

**Figure 5:**
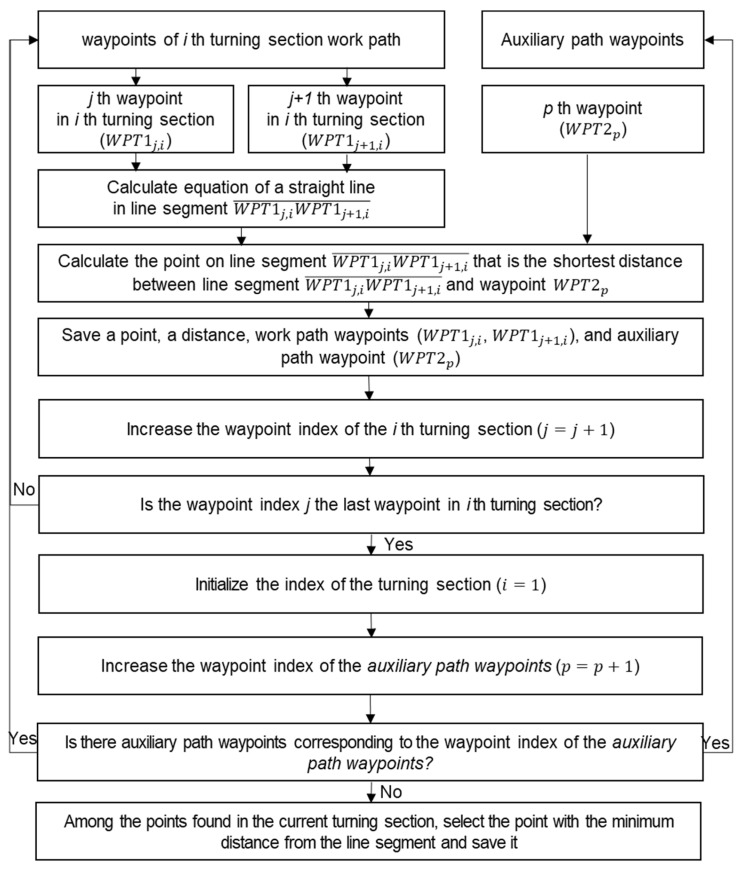
The distance-based intersection points search process.

After completing the line-segment-based intersection point search for all turning sections, the distance-based intersection point search, which was the second method of intersection points search, was performed for turning sections where intersection points did not exist. The distance-based intersection point search was used to find points where the distance between a line segment created by adjacent waypoints in a turning section work path and an auxiliary path waypoint was minimal. Figure 5 shows the distance-based intersection point search process.

The last step in the orchard passage map data generation was to create and save waypoints for work passages, location points for all orchard passages, network data, and spraying working methods using the waypoints of the working and auxiliary paths and intersection points. The waypoints for work passages were created by sequentially arranging location data along the movement path, from the start point to the end point of the work passage. This allowed the speed sprayer to continuously drive through all work passages. The location points for all orchard passages were stored, including the coordinates of work path waypoints, auxiliary path waypoints, and created intersection points. 

The stored network data indicated whether or not there was a direct connection between location points. In the network data, if there was a direct connection, the distance value between points was stored, and if there was no connection, an infinity value was stored. 

The spraying working methods were work methods (e.g., spray direction, engine RPM, whether to blow, etc.) that the speed sprayer should perform at the waypoints of work passages. To accomplish this, first, it was necessary to distinguish straight sections and turning sections using the angle between two straight lines created by adjacent waypoints in work passages. Next, the user created spraying working methods by directly entering the speed sprayer’s work methods in the straight sections and turning sections.

### 2.2. Autonomous Driving Path Generation

The autonomous driving path consisted of waypoints and the working method at the waypoints and was created using the orchard passage map data according to the operating scenario for the speed sprayer. The sprayer operating scenario consisted of four parts: return point registration, autonomous driving to the starting point of work, autonomous-driving-based spraying operation, and autonomous driving to the return point. 

In the return point registration step, the user manually moved the speed sprayer to the location near the orchard passage where it would start autonomous driving and saved the coordinates of the location. The saved location coordinates were the return point, which was the starting point of the autonomously driven speed sprayer and the point of return after completing its spraying operation. The return point was registered only if it was within a certain distance from the orchard passage on the orchard map. 

The purpose of the autonomous driving to the starting point of work step was to create an autonomous driving path from the current location to the work starting point. The work starting point was the first waypoint of the work passages or the break point of a previous autonomous-driving-based spraying operation. The method of creating an autonomous driving path to the work starting point was as follows. 

First, the starting and ending points were defined as the current speed sprayer location and the work starting point location, respectively. The ending point was set as a stopping point if the previous autonomous-driving-based spraying operation had not been completed; otherwise, it was set as the first waypoint of work passages. The second step was to update the orchard passage map data. To do this, it was first checked whether the starting and ending points were included in the location points for all orchard passages. If there was a starting and/or ending point that was not included in the location points for all orchard passages, the point on the orchard path with the shortest distance from the starting and/or ending point that was not included in the location points was calculated. Then, the location points for all orchard passages and the network data were updated using the coordinates of starting and/or ending points that were not included in the location points and the coordinates of the point that had the shortest distance from the starting and/or ending point. In the third step, the path with the shortest distance from the starting point to the ending point was found through Dijkstra’s algorithm using the network data. Finally, the shortest path from the starting point to the ending point was used to generate waypoints and the speed sprayer’s work methods for autonomous driving operations.

The autonomous-driving-based spraying operation consisted of an autonomous driving path with spraying working methods from the work starting point to the last waypoint of the work passages, while autonomously driving. When the speed sprayer was unable to perform an autonomous-driving-based operation or stopped performing autonomous driving due to the lack of battery/liquid chemical, the speed sprayer moved to the return point by autonomous driving. For autonomous driving to the return point, the starting and ending points of the autonomous driving path were defined as the current speed sprayer location and the return point location, respectively. The path creation method was the same as the process used for creating an autonomous driving path to the starting point of work.

## 3. Test Results

### 3.1. Test Platform

**Figure 6:**
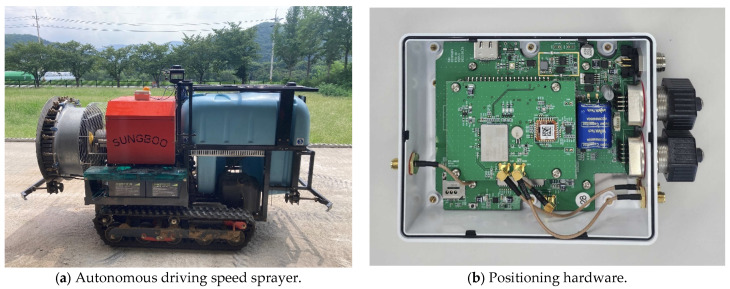
Autonomous driving speed sprayer and positioning hardware used in the test.

To verify the proposed methods, the study used a prototype of an autonomous driving speed sprayer manufactured by the Sungboo Industry Company, shown in Figure 6a. It was a crawler-type vehicle that drove on electric traction. The external dimensions of the autonomous driving speed sprayer were 2.183 m (length) × 1.300 m (width). For detailed specifications of the autonomous driving speed sprayer, please refer to [13]. The positioning hardware for autonomous driving operation was mounted on the top of the middle of the speed sprayer. Two GNSS antennas were mounted on the top of the positioning hardware and the top front of the speed sprayer, respectively. The positioning hardware (Figure 6b) consisted of an RTK-GNSS module, a motion sensor module, an embedded board, and an LTE module. The RTK-GNSS module used a unicore’s UM982 [14], which is a multi-frequency high precision positioning and heading module that uses GPS (Global Positioning System), QZSS (Quasi-Zenith Satellite System), GLONASS (GLObal Navigation Satellite System), BeiDou, and Galileo and supports the MB (Moving Baseline) RTK technique. The motion sensor using Xsens’s MTi-1 [15] contained a three-axis accelerometer, a three-axis gyroscope, and a three-axis magnetometer. The in-run bias stability of the gyroscope in the motion sensor was 10 °/h. The embedded board was the raspberry pi 4 model, which was used to operate the algorithms related to autonomous driving including positioning, orchard passage map data generation, autonomous driving path generation, and autonomous driving. The positioning algorithm used an MB RTK/Motion sensor integrated positioning algorithm [13], which could provide three-dimensional position, velocity, and attitude. 

### 3.2. Test Results of Orchard Passage Map Data Generation

**Figure 7:**
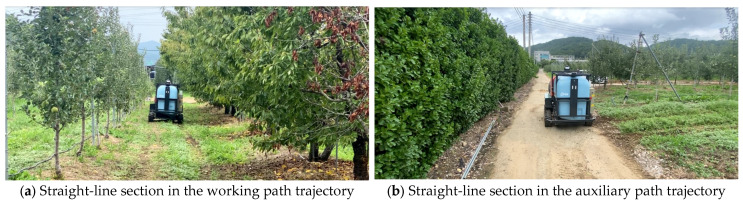
View of the orchards.

The individual path generation test was conducted at an apple farm (Gunwi, Korea) in August 2023. To test the individual path generation process, location data were acquired by manually driving the autonomous driving speed sprayer at a speed of less than 3 km/h using a wireless remote controller. The location data were geodetic coordinates (latitude, longitude, ellipsoidal height) provided at 0.5 s intervals by the MB RTK/Motion sensor integrated positioning algorithm in the positioning hardware. The trajectory of the working path included four straight-line sections and three curved sections. Apple trees were planted on the left and right sides of the straight section. The trajectory of the auxiliary path included one straight-line section. Figure 7 shows the view of the straight-line section in the working path and the auxiliary path. The minimum distance between waypoints, which are options for generating individual paths, and the angle between straight lines were set to 0.5 m and 10°, respectively.

**Figure 8:**
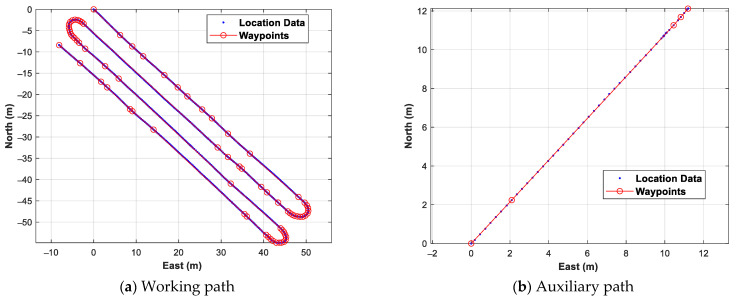
Individual path generation test results.

Figure 8 shows the location data obtained by manual driving and the waypoints created through the individual path generation, expressed using a north, east, and down (NED) relative coordinate system with the origin at the first location data. For the working path trajectory, 75 waypoints were created using 946 location data. The range of the distance between adjacent waypoints in the working path trajectory was from 0.52 m to 42.92 m. It can be seen that the distance between adjacent waypoints is long on a straight path, and the turning section is narrow. For the auxiliary path trajectory, five waypoints were created using 128 location data, and the range of distance between adjacent waypoints was from 0.57 m to 12.30 m. Based on the straight line created as adjacent waypoints, the maximum fitting errors of the working path and the auxiliary path were 0.09 m and 0.03 m, respectively. The RMS values of the fitting errors of the working path and the auxiliary path were 0.02 m and 0.01 m, respectively. As can be seen from the above results, the waypoints generated through the proposed individual path generation method were considered to reflect the driving path well.

**Figure 9:**
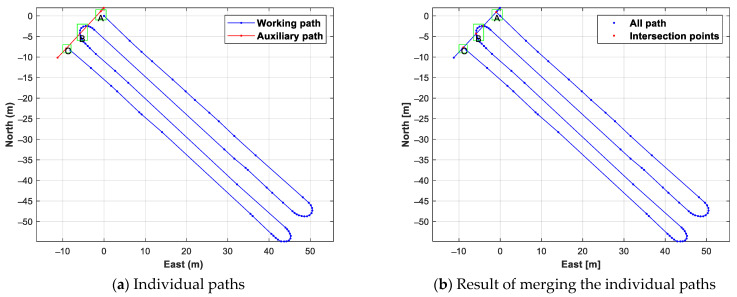
The result of merging the entered individual paths and the individual paths. A, B, and C marked with green squares in the figure are the intersection area of working path and auxiliary path.

**Figure 10:**
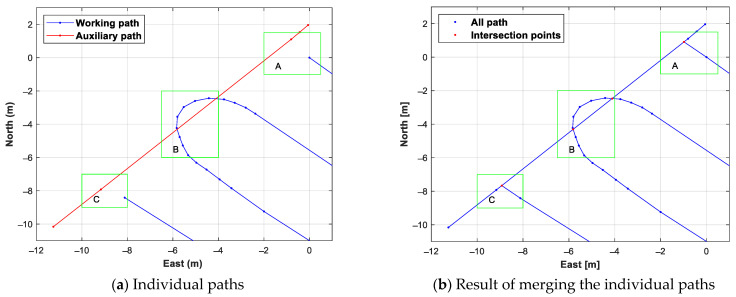
Individual paths merge result with enlarged merge area. A, B, and C marked with green squares in the figure are the intersection area of working path and auxiliary path.

Figure 9 shows the results of searching for intersection points of the work path and auxiliary path, using the proposed intersection points search method, and merging them. The intersection points search was conducted in a total of three sections, and an enlarged intersection search section is shown in Figure 10. In section B in Figure 9 and Figure 10, two intersection points were created through the line-segment-based intersection point search method, where the straight line created by the adjacent waypoints of the work path and the straight line created by the adjacent waypoints of the auxiliary path intersected. Since the work path and the auxiliary path did not intersect in sections A and C, the two waypoints with the shortest distance straight line created by the waypoint of the work path and the waypoint of the auxiliary path were connected through the distance-based intersection point search method. 

The above results confirmed that the proposed intersection point search method could merge individual paths consisting of waypoints. By merging the work path and auxiliary path in the intersection points search, two new waypoints on the work path were created, increasing the total number of work path waypoints from 75 to 77. And the numbers of points and line segments for all orchard passages were 84 and 86, respectively.

**Figure 11:**
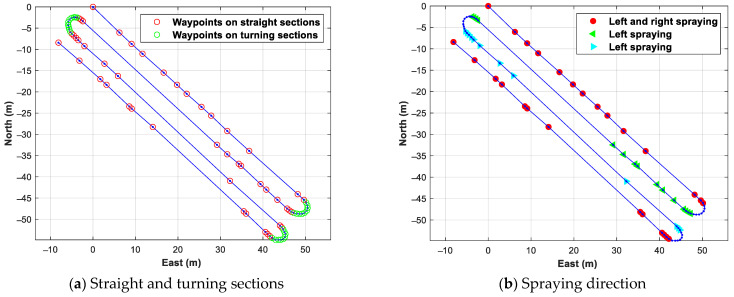
Result of defining straight and turning sections and specifying spraying direction in straight sections.

The final results of defining straight sections and turning sections, using the proposed method to specify work methods at waypoints of the work path, are shown in Figure 11a. The numbers of straight sections and turning sections were four and three, respectively, and as can be seen from Figure 11a, they were divided according to the path geometry. Using the waypoints in each straight section, the spraying direction for the first and fourth straight sections was specified on both sides, and the spraying direction for the second and third straight sections was specified on one side. The result of specifying the spraying direction is shown in Figure 10a. As can be seen from this result, it was found that it was possible to distinguish between straight and turning sections and define work methods corresponding to each section.

### 3.3. Test Results of Autonomous Driving Path Generation

**Figure 12:**
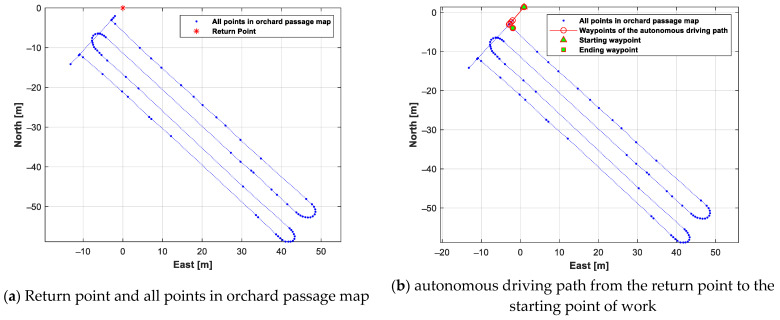
Results of registering the return point and generating autonomous driving path from the return point to the starting point of work.

An autonomous driving path generation test was conducted based on an autonomous driving operation scenario, using the orchard passage map generated by the orchard passage map data generation test. The work starting point of the speed sprayer was assumed to be the first waypoint of the work passages. The result of defining the return point and then generating an autonomous driving path from the return point to the work starting point is shown in Figure 12. As can be seen in Figure 12, the autonomous driving path to the work starting point was generated through the shortest path. 

**Figure 13:**
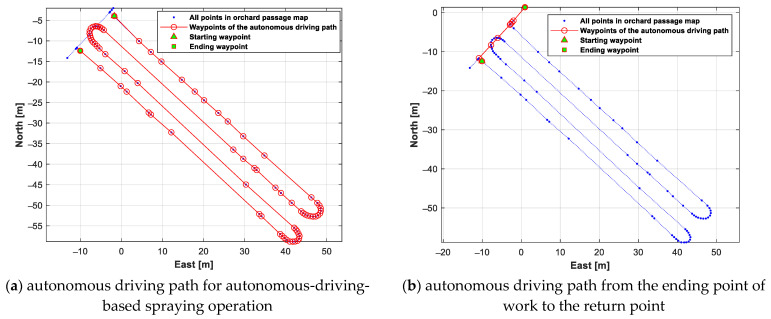
Result of generating an autonomous driving path for autonomous-driving-based spraying operation and generating an autonomous driving path from the ending point of work to the return point.

Figure 13a shows the autonomous driving path of the autonomous-driving-based spraying operation. Figure 13b shows the autonomous driving path to the return point after completing the autonomous spraying operation. As can be seen in Figure 13b, the autonomous driving path to the return point was the shortest path.

**Figure 14:**
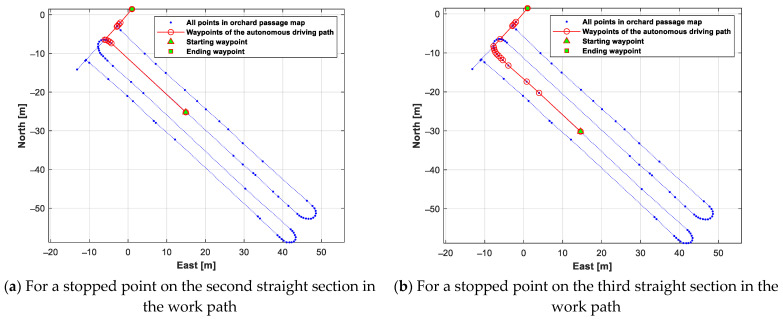
Autonomous driving paths generated from each stopped point to the return point.

**Figure 15:**
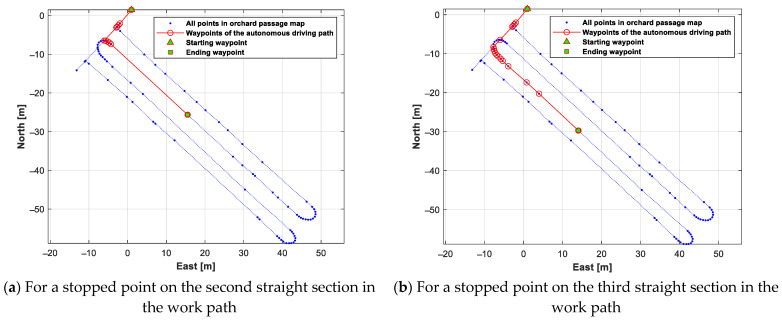
Autonomous driving paths generated from the return point to each stopped point.

A test was conducted to generate an autonomous driving path from the stopped point to the return point when the autonomous-driving-based spraying operation was stopped due to the lack of battery/liquid chemical. For this test, two stopped points were set, on the second and third straight sections of the work path. The generated autonomous driving path from each stopped point to the return point is shown in Figure 14. Figure 15 shows the generated autonomous driving path from the return point to each work starting point (the stopped point of the autonomous-driving-based spraying operation). 

**Figure 16:**
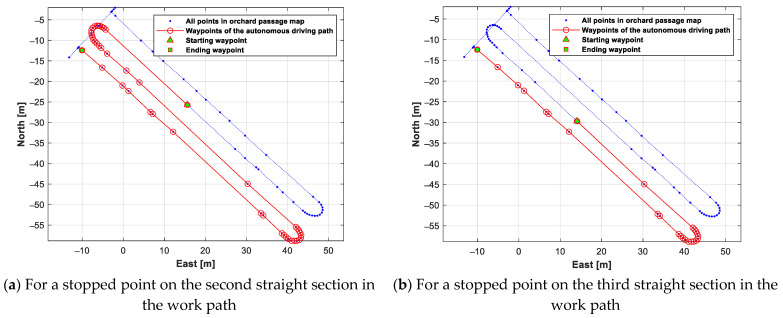
An autonomous driving path generated for autonomous-driving-based spraying work from each work starting point.

The generated autonomous driving path for autonomous-driving-based spraying work from each work starting point (the stopped point in the autonomous-driving-based spraying operation) is shown in Figure 16. The above results indicate that an autonomous driving path suitable for the autonomous driving speed sprayer operating scenario can be generated using the orchard passage map data prepared with the method proposed in this study.

## 4. Conclusions and Future Work

In this study, to contribute to the commercialization of self-driving speed sprayers, we presented a method for generating an orchard passage map using location data obtained from a positioning sensor and a method for generating an autonomous driving path using the same. The proposed method for generating an orchard passage map consisted of an individual path generation method and a method of merging the generated individual paths. Individual paths were created using location data acquired by manually driving the speed sprayer on the work path where the work was to be performed and the auxiliary path, which was an orchard passage excluding the work path. Then, the orchard passage map was created by connecting the work path and the auxiliary path using an intersection point search process and an individual path merging method. In addition, straight sections and turning sections for the work path were distinguished, to allow the work method for the work section to be defined for the speed sprayer. 

A method was also presented to enable autonomous driving using an orchard passage map, by generating an autonomous driving path based on an operating scenario. The method was then used to conduct tests in an actual orchard environment. The orchard passage map generation test results demonstrated that a connecting relationship between location points could be defined for orchard passages using the proposed method. The test to create an autonomous driving path based on an operating scenario confirmed that the shortest autonomous driving path could be appropriately generated using an orchard passage map.

The proposed method in this study is useful for the related research in that it can generate an orchard passage map using only location data and use this to generate an autonomous driving path suitable for the operation scenario of the speed sprayer. In addition, using the proposed methods in this study can make it possible to create an orchard passage map and perform autonomous driving operations using autonomous agricultural machinery equipped with only positioning sensors, without having to install additional sensors such as LiDAR and cameras. Therefore, it is considered that using the proposed method can contribute to the dissemination and utilization of autonomous agricultural machinery using only positioning sensors, which are currently under development. However, the proposed method in this study has the following limitations: First, it is difficult to apply it to orchards with a large area because it is necessary to obtain manually driven location data for the orchard passage. Additionally, since GNSS positioning sensors are affected by the satellite reception environment, the precise location cannot be determined in leafy areas, which may result in errors when generating passage maps. Since the generated path consists of points, information such as the width of the passage cannot be known. Therefore, in future research, to solve the above problems, we will develop a method to generate an orchard map by fusing various information such as aerial images, LIDAR, and vision cameras used in route generation and compare the pros and cons of each type of fused information. We plan to verify the proposed methods in various other types of orchards, find problems, and update them. In addition, using the orchard map created through the method proposed in this study, we will conduct additional research on path generation methods for autonomous driving operation of various agricultural machines such as weeders and transport robots.

## Data Availability

Data are contained within the article.
